# Effect of Hypohydration on Peripheral and Corticospinal Excitability and Voluntary Activation

**DOI:** 10.1371/journal.pone.0077004

**Published:** 2013-10-02

**Authors:** Joanna L. Bowtell, Gareth Avenell, Steven P. Hunter, Katya N. Mileva

**Affiliations:** 1 Sport and Exercise Science, London South Bank University, London, United Kingdom; 2 Sport and Health Sciences, Exeter University, Exeter, United Kingdom; University of Sydney, Australia

## Abstract

We investigated whether altered peripheral and/or corticospinal excitatory output and voluntary activation are implicated in hypohydration-induced reductions in muscle isometric and isokinetic (90°.s^−1^) strength. Nine male athletes completed two trials (hypohydrated, euhydrated) comprising 90 min cycling at 40°C, with body weight losses replaced in euhydrated trial. Peripheral nerve and transcranial magnetic stimulations were applied during voluntary contractions pre- and 40 min post-exercise to quantify voluntary activation and peripheral (M-wave) and corticospinal (motor evoked potential) evoked responses in m. vastus medialis. Both maximum isometric (−15.3±3.1 vs −5.4±3.5%) and isokinetic eccentric (−24.8±4.6 vs −7.3±7.2%) torque decreased to a greater extent in hypohydrated than euhydrated trials (p<0.05). Half relaxation time of the twitch evoked by peripheral nerve stimulation during maximal contractions increased after exercise in the hypohydrated (21.8±9.3%) but stayed constant in the euhydrated (1.6±10.7%; p = 0.017) condition. M-wave amplitude during maximum voluntary contraction increased after exercise in the heat in hypohydrated (10.7±18.0%) but decreased in euhydrated condition (−17.4±16.9%; p = 0.067). Neither peripheral nor cortical voluntary activation were significantly different between conditions. Motor evoked potential amplitude increased similarly in both conditions (hypohydrated: 25.7±28.5%; euhydrated: 52.9±33.5%) and was accompanied by lengthening of the cortical silent period in euhydrated but not hypohydrated condition (p = 0.019). Different neural strategies seem to be adopted to regulate neural drive in the two conditions, with increases in inhibitory input of either intracortical or corticospinal origin during the euhydrated trial. Such changes were absent in the hypohydrated condition, yet voluntary activation was similar to the euhydrated condition, perhaps due to smaller increases in excitatory drive rather than increased inhibition. Despite this maximal isometric and eccentric strength were impaired in the hypohydrated condition. The increase in peripheral muscle excitability evident in the hypohydrated condition was not sufficient to preserve performance in the face of reduced muscle contractility or impaired excitation-contraction coupling.

## Introduction

It is well accepted that endurance performance is impaired once body water losses exceed 2% of body weight (for recent review see [1]). However, the literature is less consistent with regard to the effects of hypohydration on maximal muscle strength and power. This is most likely due to the significant variation in: methods employed to induce hypohydration (passive vs active heat exposure, fluid restriction, diuretics), and outcome measures used to assess muscle strength and power (isometric and isokinetic peak torque and vertical jump height). These factors also make it difficult to isolate the effects of hypohydration. For instance, both prior exercise and passive hyperthermia [2] impair performance irrespective of hydration status. Vertical jump performance which is dependent upon the power: weight ratio will improve due to the hypohydration related loss of body weight unless, as demonstrated by Cheuvront et al. [3], it is matched by a decline in power. Judelson et al. [4] comprehensively reviewed the published data and after identifying papers that seemed to avoid these confounding factors, concluded that a 3–4% hypohydration induced ∼2% loss in muscle strength. More recently, Hayes and Morse [5] conducted a dose-response study employing exercise in the heat to induce hypohydration and found that knee extensor maximum force production was significantly impaired once hypohydration reached 2.6% body weight loss with an 8.5 and 8.2% reduction in maximum torque during isometric and slow isokinetic contractions (30°.s^−1^), respectively. However, force production was not impaired during fast isokinetic contractions (120°.s^−1^) even when hypohydration reached 3.9% body weight loss.

These effects have been attributed to altered neuromuscular function but there is only relatively limited empirical evidence to support these assertions [6], [7], [8]. Neuromuscular function is dependent on peripheral factors such as excitation-contraction coupling, neuromuscular junction transmission and motor axon excitability; as well as central factors including motor unit recruitment and firing rates, changes in excitability or responsiveness of spinal motoneurons to synaptic input, insufficient or inefficient output from the motor cortex circuitry and the influence of higher centres. To date the relative contribution of these different levels within the hierarchy of motor control to any hypohydration-induced deterioration in neuromuscular function has not been systematically studied. Vallier et al. [9] found that maximum voluntary isometric knee extensor force production and EMG amplitude decreased after 3 h cycling at 60% VO_2_max irrespective of whether fluid was ingested or not, however the reduction in mean frequency of the EMG power spectrum was attenuated by fluid ingestion. This could be attributed to peripheral effects of fluid ingestion on muscle fibre ionic balance, membrane potentials and hence action potential conduction velocity [10]. However these findings are not consistent across studies, with others reporting contradictory findings [6], [7], [11].

The combined effects of hypohydration and hyperthermia have been explored using peripheral nerve and/or muscle electrical stimulation which allows differentiation between the contribution of central and peripheral elements to alterations in neuromuscular function. Periard et al. [12] found that both passive and active hyperthermia (39.5°C core temperature) accompanied by 1% hypohydration resulted in reduced voluntary activation but this could account for less than 42% of the total reduction in maximum voluntary contraction (MVC) force. This implies significant peripheral contributions to fatigue induced by heat and hypohydration. The 3.9% body weight loss induced by cycling at 60% VO_2_max in 36°C for 2 h without fluid ingestion resulted in an 11% reduction in MVC force in comparison to a smaller 6% reduction in force where euhydration was maintained by water ingestion [13]. However voluntary activation, assessed by electrical stimulation of the muscle, declined similarly by 4–5% in both conditions and could not therefore account for the augmented reduction in MVC force during the no-fluid trial. Unfortunately, neither twitch time parameters such as time to peak twitch or half relaxation time nor compound motor action potential data are available in either study. Nor to our knowledge have any studies employing TMS in combination with PNS been conducted to allow the contribution of central and peripheral excitatory output to be explored.

Certainly, studies in which hyperthermia is induced either passively [2], [14], [15], [16] or actively [12], [13] but where hydration status is maintained by water ingestion suggest a significant contribution of spinal and peripheral components to fatigue. The reduction in MVC caused by passive or active hyperthermia without hypohydration is accompanied by reduced voluntary activation as well as reduced H-reflex and M-wave amplitudes implicating altered spinal and peripheral excitatory output, respectively (for review see [17]). To our knowledge there is only one published study in which the effects of hypohydration on cortical excitability have been directly investigated. Montain & Tharion [18] explored the effect of 4% passive hypohydration on somatosensory potentials evoked by peripheral nerve stimulation during repetitive isometric thumb adduction and found some indication of altered afferent signal processing. However in view of the task and muscle specificity of changes in cortical and corticospinal excitability [19], it is not clear whether these findings could be extrapolated to larger muscle groups; nor what their relevance might be for motor function. Hence the purpose of the present study was to investigate whether the decline in muscle strength induced by hypohydration is accompanied by altered excitatory output of the peripheral and corticospinal pathways. Hypohydration was induced by exercise in the heat but in an attempt to exclude the confounding influence of hyperthermia, the post-exercise testing was conducted after 40 min recovery to allow core and skin temperature to return to pre-exercise levels.

## Methods

### Ethical Approval

Nine male well-trained taekwondo athletes (age: 23±5 y; height: 1.77±0.04 m; weight: 68.2±1.2 kg) took part in this study, which was approved by the London South Bank University Research Ethics Committee and conducted in accordance with the Declaration of Helsinki. After being informed verbally and in writing of the experimental procedures and associated risks, all participants completed a medical health questionnaire and provided written informed consent to the experimental procedures. All participants were in good health and had no known history of cardiovascular, metabolic, neurological or motor disorder.

### Protocol

Each participant attended the laboratory on two occasions separated by at least one week, in order to complete the two main trials: euhydrated (Eu) and hypohydrated (Hy). During a preliminary visit, participants were familiarised with the experimental protocol and techniques, including the sensation of the peripheral nerve and transcranial magnetic stimulation, and performing the maximal isometric and isokinetic contractions. Trials were allocated by systematic rotation to counterbalance the design for trial order. Upon arrival at the laboratory participants swallowed the telemetry Cortemp pill (HQ Inc, Florida, USA), to monitor core temperature, with 200 ml water. Participants were then asked to empty their bladders and body weight (kg) and height (m) (Seca, Hamburg, Germany) were measured, and capillary blood samples were taken and analysed for haemoglobin (Haemocue, Angelholm, Sweden) and haematocrit (Hawksley, Sussex, UK). Changes in plasma volume were calculated using the formulae of Dill et al. [20].

**Figure 1:**
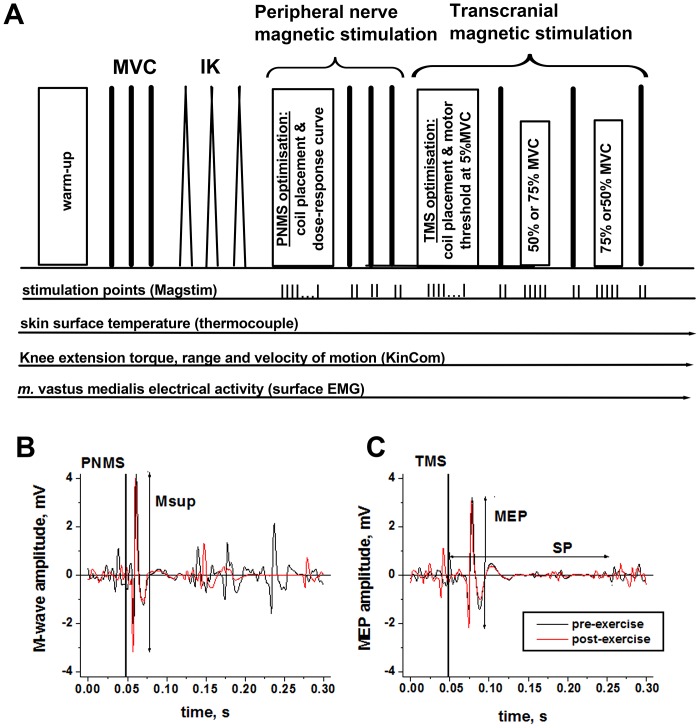
Experimental protocol. Schematic diagram of the experimental procedures and the recorded signals; MVC – maximal voluntary contraction; IK – isokinetic contraction (**A**); examples of the maximum compound muscle potentials (M-wave) evoked by peripheral nerve magnetic stimulation (PNMS) delivered to the femoral nerve (**B**) and the motor evoked potentials (MEP) evoked by transcranial magnetic stimulation (TMS) delivered to the motor cortex (**C**). The responses were recorded from the vastus medialis (VM) muscle of a representative participant during MVC pre and post-exercise; Msup – amplitude of the maximal M-wave; MEP – amplitude of the motor evoked potentials; SP – duration of the cortical silent period following the MEP.

Participants were then seated in the isokinetic dynamometer (KinCom, Tennessee, USA) for attachment of the EMG electrode over m. vastus medialis (VM) of the right leg, and a thermocouple to measure skin temperature. Participants were placed in a semi-supine position on the isokinetic dynamometer with the knee at 90° flexion, and straps were fixed around the right leg and hips. The semi-supine positioning allowed better access to the superficial femoral nerve for the peripheral magnetic stimulations [21]. Participants completed 3 sets of 10 isokinetic (30°.s^−1^) concentric/eccentric knee extension contractions at approximately 50% of maximal effort in order to warm-up (Fig. 1).

Participants then completed 3 brief (3 s) maximal voluntary isometric knee extension contractions at a 90° knee angle, followed by 3 maximal concentric/eccentric isokinetic knee extension contractions at 90°.s^−1^; with 2 min rest between contractions. Five single pulse peripheral magnetic stimulations were delivered to the femoral nerve at 100% stimulator output at rest and then a stimulation pulse was delivered during and 2 s after each of 3 brief (3 s) MVC to obtain a potentiated twitch. Participants then received 5 suprathreshold transcranial magnetic stimulations at slightly pre-activated muscle (5%MVC) and subsequently performed a brief (3 s) maximal isometric contraction. This was followed by two sets of 5 brief isometric submaximal knee extension contractions, each followed by an MVC after 1 min rest. Participants were asked to bring the torque back to their target levels as quickly as possible after stimulation. Submaximal contractions were performed at 50 and 75% MVC with visual feedback of the target torque level provided to allow participants to regulate their effort. There was a 2 min rest between the MVC and the subsequent submaximal contractions. Throughout this protocol, participants received suprathreshold transcranial magnetic stimulations, which were delivered during each of the submaximal and maximal contractions to allow calculation of the estimated resting twitch. After completion of the stimulation protocols the position of the electrodes and thermocouple was marked with indelible pen onto the skin, and then the sensors were removed.

Participants then entered the environmental chamber and completed 6×15 min bouts of cycling at 80% age predicted heart rate maximum (HR_max_) at 40°C and 50% humidity. Nude body weight of participants was measured after towelling down at the end of each 15 min block of exercise, and during the euhydrated trial participants consumed a volume of fluid (lemon flavoured saline, 20 mM Na) equal to the weight loss. In the hypohydrated trial, no fluid was consumed. Core body temperature and heart rate were monitored at 5 min intervals throughout, and if core body temperature exceeded 39.5°C, participants were removed from the chamber and placed in a cool environment until core temperature returned below 39°C.

Participants were weighed 5 min after completion of the final 15 min exercise bout, and a final drink consumed in the euhydrated trial. Participants then returned to the laboratory and the sensors were re-attached at the marked locations. The stimulation protocol was repeated 20 min after leaving the chamber. Capillary blood samples were taken after the protocol (∼60 min after leaving the environmental chamber) and analysed for haemoglobin and haematocrit.

### Stimulations

Two forms of magnetic stimulation were used: peripheral stimulation of the femoral nerve (PNMS) and transcranial stimulation of the motor cortex (TMS). Motor evoked potentials (MEPs) in the knee extensor muscles were elicited by TMS of the contralateral motor cortical leg area provided by a Magstim 200 stimulator (Magstim Co Ltd,UK). Single pulses of 100 µs duration and up to 2 T intensity were delivered to the motor cortex through 110° double cone coil (9 cm diameter each, type P/N 9902-00). The optimal coil position was determined by moving the coil along a virtual grid lateral and posterior to the vertex (±3 cm) with a resolution of 1 cm until reaching a position at which maximal MEP amplitude in the slightly pre-activated VM (5%MVC) was produced with minimal stimulation intensity. To optimise the stimulation intensity, the stimulus-response curve was constructed for each subject by systematically varying the TMS intensity. The active motor threshold (aMT) was defined as the lowest TMS intensity eliciting a MEP in the VM muscle of minimum 200 µV peak-to-peak amplitude in at least 50% of the stimulations during a knee extension contraction at 5% MVC [22]. MEPs of this magnitude were clearly discernible from the voluntary VM EMG activity for all participants since exceeding background peak-to-peak EMG amplitude by at least 2SD. aMT was verified after environmental chamber exposure but no change occurred for any participant in either trial. The stimulation intensity during the exercise protocols was then set to 120% aMT.

Peripheral stimulation of the femoral nerve was achieved using a Magstim 200 stimulator equipped with a 70 mm figure of eight polyurethane coated coil capable of producing a maximum output of 2.2 T. The stimulating coil head was positioned high in the femoral triangle just lateral to the femoral artery. The best location producing the maximal twitch was identified with systematic minor position adjustments and then marked on the skin for the remainder of the experiment. All stimulations delivered during the exercise protocols were at 100% stimulator output. To verify that the PNMS at 100% stimulator output was of sufficient intensity to evoke maximal twitch and M-wave amplitude a dose response curve was constructed by delivering 3 stimulations at each 80, 85, 90, 95 and 100% stimulator output, both before and after environmental chamber exposure. Peak twitch increased by only 5.5±1.4% when stimulator intensity was increased from 95 to 100%. Verges et al. [21] recently verified that the fatigue-induced changes in force and M-wave were very similar whether quantified by single electrical or magnetic stimulation of the femoral nerve.

### Data Collection

Voluntary EMG level, compound motor action potentials (M-waves) and motor evoked potentials (MEPs) were recorded from VM muscle of the right leg using surface active bipolar bar EMG electrodes (99.9% Ag, 10 mm length, 1 mm width, 10 mm pole spacing, CMRR >80 dB, model DE2.1, DelSys Inc, Boston, MA). The electrode was placed over the muscle belly at the recommended optimal site for surface EMG recording from VM muscle [23]. The ground electrode was placed over the patella of the right leg. Double-sided adhesive interfaces and a hypoallergic medical tape were used to ensure the stability of the contact between the EMG sensor and the skin. The skin area underneath the EMG electrode was shaved, then exfoliated with abrasive gel, and cleaned with ethyl propanol to minimize the skin impedance. The EMG signal was preamplified (×1000) and band-pass filtered between 20–450 Hz at the source (Bagnoli-8, DelSys Inc, Boston, MA), then transferred to a computer with a sampling frequency of 2 kHz and high-pass filtered at 10 Hz. Special care was taken during the experiments to minimize the contamination of the EMG signal with movement artefacts and interferences from external electromagnetic sources.

Skin temperature was continuously measured using a thermocouple, taped to the leg over the belly of m. vastus lateralis and digitised at 50 Hz sampling frequency. A welded tip thermocouple (Type K, PTFE insulated, nickel-chromium/nickel-aluminium) was used in conjunction with an automatic cold junction compensation amplifier giving 10 mV per °C output.

The knee extension torque produced during the tests as well as knee joint range of motion and angular velocity were measured using an isokinetic dynamometer (KinCom, Tennessee, USA). Dynamometry data (joint angular displacement, angular velocity, torque) were sampled with a frequency of 200 Hz and recorded continuously and synchronously with the EMG and skin temperature signals in a PC via an A/D converter (CED 1401power, Cambridge, UK), using Spike2 data acquisition software (CED, Cambridge, UK) with 16 bit resolution.

### Data analysis

Data were analysed off-line using custom written scripts developed in Spike2 ver.6 software (CED, Cambridge, UK).

#### Peak Voluntary Torque

The maximum isometric knee extension torque (MVC, Nm) was evaluated before and after the fatiguing protocol and calculated as the average value over a 1-second period around the plateau level of the highest torque achieved in the 3 non-stimulated maximal voluntary isometric contractions completed at each measurement time point. Peak isokinetic concentric and eccentric torques were identified in each of the three maximal isokinetic (IK) contractions completed at each measurement time point. The maximum value achieved for each parameter at each time point is presented in the results. Voluntary torque (VT, Nm) during the contractions in which PNMS or TMS were applied was calculated as the mean torque level achieved during the 50 ms preceding the stimulation pulse delivery.

#### Excitatory Output

MEP and M-waves were non-polyphasic as indicated by the example traces provided in Figure 1, hence peak to peak amplitude data rather than area are presented. The peak-to-peak amplitude of the maximal M -wave was quantified pre and post exercise in the heat at rest (Mmax) and during MVC (Msup) to reflect the excitability of the peripheral circuitry (motor axon, neuromuscular junction, and muscle fibre membrane). The corticospinal excitatory output to VM was quantified at each stimulation point by the MEP peak-to-peak amplitude normalised to the M-wave amplitude acquired at the corresponding activation level and time point. The excitability of the intracortical inhibitory pathways was assessed by the duration of the silent period (SP, ms) measured between the stimulation point and the time when the muscle activation returned to the prestimulation voluntary EMG level [24]. This was assessed by calculating the RMS EMG amplitude in the 500 ms preceding the delivery of each TMS pulse.

#### Peripheral Contractility

The peripheral contractility during maximal voluntary effort was quantified by the maximal muscle relaxation rate (RR, 1/s) which occurs during the TMS evoked silent period. RR was identified from the time derivative of the torque signal and normalised to the overall torque (sTw+VT), where, the superimposed twitch (sTw, Nm) was calculated as the peak torque increment evoked by TMS above the voluntary torque (VT) level. Peripheral contractility during MVC was also assessed from the half relaxation time measured from the twitch evoked by peripheral nerve magnetic stimulation.

The muscle contractility at rest was evaluated by the estimated resting twitch (eRTw, Nm) calculated as the y-intercept of the linear regression between the sTw elicited by TMS and the VT registered during each brief voluntary contraction of 50, 75 and 100% MVC [25]. The size of the potentiated twitch (pTw) evoked by PNMS delivered to the resting VM immediately after the MVCs was also used to quantify peripheral contractility changes due to the investigated conditions.

#### Voluntary Activation

The efficiency of the excitatory output was assessed by the voluntary activation of the motoneuron pool (VA,%), calculated using the standard twitch interpolation equation VA = (1-sTw/eRTw)*100,% [16]. Voluntary activation was also assessed using the superimposed (sTw) and the potentiated (pTw) twitch responses to peripheral nerve magnetic stimulation: VA = (1-sTw/pTw)*100,%. In addition, the peak superimposed twitch elicited by stimulation during the MVC and normalised to the peak voluntary torque achieved prior to stimulation (sTw/VT,%) was used to investigate potential contribution of spinal and supraspinal mechanisms.

### Statistical analyses

Data are presented as mean ± SEM and were statistically analysed by repeated measures two way ANOVA (condition: euhydrated and hypohydrated versus time: pre and post exercise in the heat). Where appropriate, the effect size statistic (η^2^) was also calculated. Intra-class correlation analysis was conducted to assess the test-retest reliability of the baseline kinetic and EMG measures using the pre-exercise data from the two visits. A one-way random-effects single measures model was applied for the torque performance parameters and one-way random-effects average measures model to calculate the intraclass correlation coefficients (ICC) for all other parameters.

The overall acceptable significance level of differences for all statistical tests was set at p<0.05. The statistical analyses were performed in SPSS ver 18 (SPSS Inc., Chicago, IL) and Origin version 6.0 (Microcal Software Inc.) package software.

## Results

### Hydration and Temperature Measures

After completing the 90 min cycling in the environmental chamber, body weight decreased by 2.8±0.1% in the hypohydrated trial and by 0.4±0.1% in the euhydrated trial (Table 1). This small fluid deficit in the euhydrated trial was matched by the volume of the final drink, consumed after leaving the chamber. After completion of the post-exercise PNMS and TMS stimulation protocol (ending ∼60 min after leaving the chamber), plasma volume had increased relative to baseline in both Eu (9.7±3.4%) and Hy (4.2±2.4%) trials, but there was no statistically significant difference between trials.

**Table 1:** Population average body weight, skin temperature and isometric and isokinetic peak torque data pre and post 90°C and 50% humidity during euhydrated (Eu) and hypohydrated (Hy) trials with intraclass correlation coefficients (ICC) provided.

Parameter	Condition	Pre-exercise	Post-exercise
Body Weight (kg)* #	Eu	68.1±1.2	68.1±1.2
	Hy	68.2±1.2	66.3±1.2
Tsk (°C)	Eu	18.7±0.2	18.1±0.2
	Hy	18.5±0.3	18.6±0.1
MVC peak torque (Nm)* #	Eu	703.1±42.6	663.5±43.8
(ICC: 0.75)	Hy	714.2±58.2	604.9±51.5
Isokinetic eccentric peak torque	Eu	672.8±6.5	595.2±57.3
(Nm)* # (ICC: 0.83)	Hy	745.3±91.0	553.0±48.1
Isokinetic concentric peak torque	Eu	417.6±36.4	327.5±34.8
(Nm)* (ICC: 0.73)	Hy	409.8±34.2	353.2±33.6

Data are mean ± SEM and n = 9;

*indicates a main effect of time and ^#^ indicates a condition x time interaction effect, both p<0.05.

**Figure 2:**
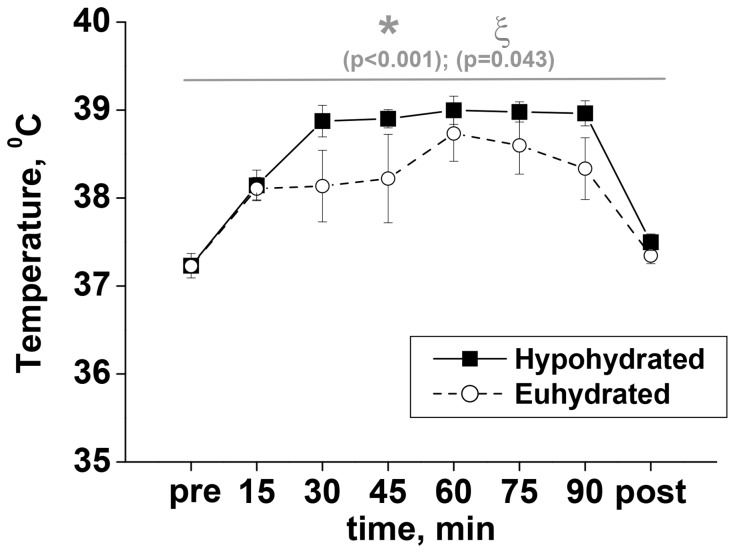
Core body temperature. Population average (±SEM, n = 9) responses measured throughout the experimental protocol completed in hypohydrated and euhydrated conditions; * - main time effect, and ξ - main condition effect.

Core body temperature increased during exercise in the heat (p<0.001), and was higher in the Hy trial for the last 30 min of exercise (p = 0.043), but there was no significant condition by time interaction (Fig. 2). Core body temperature had returned close to baseline values (37.2±0.1°C) prior to commencing the post-exercise PNMS and TMS stimulation protocols, and was not different between Eu (37.3±0.1°C) and Hy (37.5±0.1°C) trials. Similarly there was no effect of either time or condition on skin temperature (Tsk) measured during the PNMS and TMS stimulation protocols (Table 1).

### Torque

Maximum isometric knee extensor torque decreased after 90 min of exercise in the heat (P = 0.007) in both trials, but to a greater extent in the Hy than Eu trial (−15.3±3.1 vs −5.4±3.5%, p = 0.026 condition × time interaction effect). Similarly peak isokinetic eccentric and concentric torque decreased after 90 min of exercise in the heat (p = 0.006, p = 0.004 respectively) in both trials, and peak eccentric torque decreased to a greater extent in the Hy trial (−24.8±4.6 vs −7.3±7.2%, p = 0.002 condition × time interaction effect; Table 1).

### Peripheral Contractility

#### Twitch Amplitude in Relaxed Muscle

The normalised estimated resting twitch (eRTw) tended to increase after exercise in the heat to a greater extent in the hypohydrated (25.0±14.6%) than euhydrated (12.0±10.8%) condition (Table 2). The normalised potentiated twitch (pTw) was not different between conditions but also tended to increase to a greater extent in the hypohydrated (19.5±10.1%) than euhydrated (5.2±8.3%) condition. However these tendencies did not achieve statistical significance (Table 2). The absolute ERTw and pTw were not affected by either exercise in the heat or condition (Table 2).

**Table 2:** Peak twitch and voluntary activation data.

Parameter	Stimulation	Trial	Pre (% MVC)	Post (% MVC)
Peak Twitch	TMS*	Eu	2.0±0.6	2.8±0.8
sTw	(ICC: 0.96)	Hy	1.7±0.6	2.6±0.7
	PNMS*	Eu	4.8±1.9	5.1±1.7
	(ICC: 0.82)	Hy	3.6±1.0	4.4±1.1
Voluntary Activation	TMS	Eu	85.5±3.1	84.4±4.2
VA	(ICC: 0.72)	Hy	90.4±2.2	84.9±3.8
	PNMS	Eu	81.4±4.2	79.1±4.2
	(ICC: 0.95)	Hy	83.1±3.7	83.1±3.2
Estimated Resting Twitch	TMS	Eu	11.8±1.5	13.1±1.6
eRTw	(ICC: 0.96)		[87.4±10.2 Nm]	[88.9±10.3 Nm]
		Hy	11.5±0.9	15.4±2.4
			[87.5±9.9 Nm]	[88.6±11.1 Nm]
Potentiated Twitch	PNMS	Eu	19.3±2.0	19.6±1.4
pTw	(ICC: 0.95)		[137.4±13.2 Nm]	[130.5±13.1 Nm]
		Hy	18.5±1.7	21.3±1.7
			[125.7±13.2 Nm]	[122.6±14.3 Nm]

Population average kinetic responses evoked by peripheral nerve (PNMS) and transcranial magnetic (TMS) stimulation: peak superimposed twitch (sTw) during maximum voluntary contraction (MVC), potentiated twitch (pTw) at rest and estimated resting twitch (eRTw) data pre and post exercise in the heat during euhydrated (Eu) and hypohydrated (Hy) trials, with intraclass correlation coefficients (ICC) provided. Data are mean ± SEM and n = 9;

*indicates a main effect of time, p<0.05.

#### Twitch Temporal Parameters

**Figure 3:**
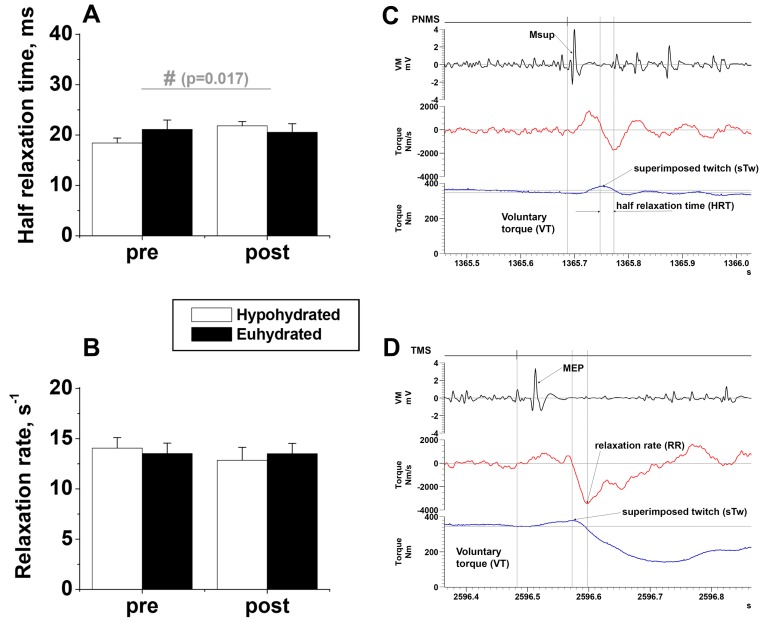
Peripheral Muscle Contractility. Half relaxation time (HRT: mean ±SEM, n = 9) of the twitch responses to peripheral nerve stimulation (**A**) and relaxation rate (RR: mean ±SEM, n = 9) observed after transcranial magnetic stimulation (**B**) during maximal voluntary contractions (MVC) completed before (pre-) and after (post-) exercise in the heat in hypohydrated and euhydrated conditions. Torque responses indicating HRT (**C**) and RR (**D**) are provided for a representative participant; # - interaction effect.

The half relaxation time (HRT) of the twitch evoked by peripheral nerve stimulation during an MVC increased after exercise in the hypohydrated condition (21.8±9.3%) whilst remaining relatively constant in the euhydrated condition (1.6±10.7%; p = 0.017, main interaction effect, Fig 3A). Similarly the twitch relaxation rate (RR) after motor cortex stimulation during an MVC tended to be slower after exercise in the heat in the hypohydrated condition (−8.9±3.0%) but increased slightly in the euhydrated condition (5.6±7.6%, Fig 3B). However these tendencies for different RR changes over time in the two conditions did not achieve statistical significance.

### Peripheral Excitability

**Figure 4:**
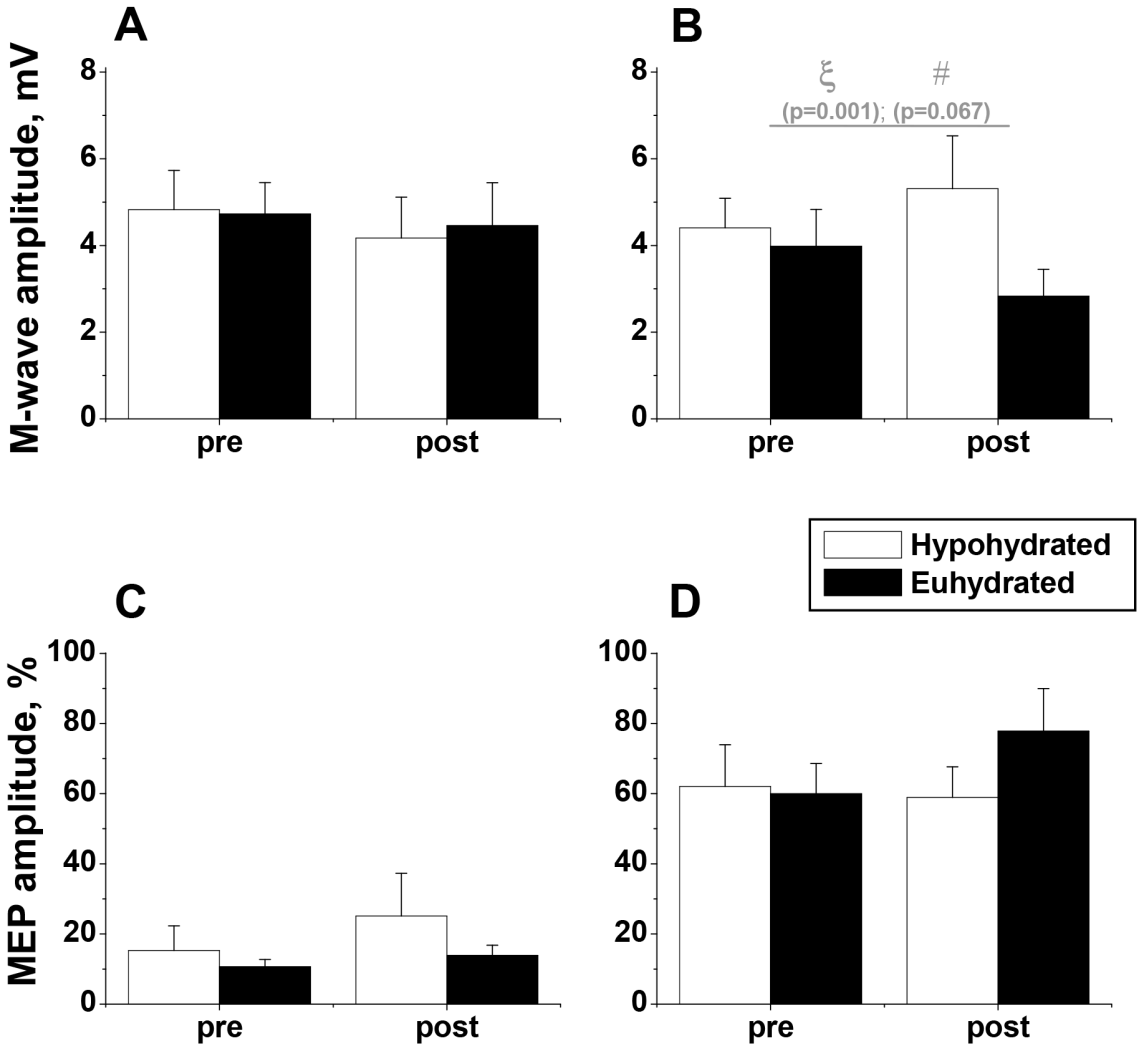
Muscle Evoked Potentials. Amplitudes of the population average (±SEM, n = 9) maximal M-wave in resting (Mmax) muscle (**A**) and in an active muscle (Msup) performing maximum voluntary contraction, MVC (**B**). Amplitudes of the motor evoked potentials (MEP) normalised to corresponding M-wave amplitude during low level contraction (**C**) and MVC (**D**). The responses were recorded before (pre) and after exercise in the heat in hypohydrated and euhydrated conditions; ξ - main condition effect, and # - interaction effect.

M-wave amplitude at rest (Mmax ICC: 0.79) was not significantly altered after exercise in the heat (Fig 4A). During maximal voluntary contraction, M-wave amplitude (Msup ICC: 0.94) was significantly higher during the hypohydrated condition (p = 0.001, main condition effect, Fig. 4B). In addition, the muscle response to nerve stimulation after the exercise in the heat tended to be different depending upon condition, with Msup amplitude increasing after exercise in the hypohydrated condition (10.7±18.0%) but decreasing during the euhydrated condition (Eu: −17.4±16.9%; p = 0.067 condition × time interaction effect; η^2^ = 0.4; Fig 4B).

### Voluntary Activation

Peripheral voluntary activation did not change after exercise in the heat but cortical voluntary activation (VA) assessed using TMS tended to decrease (Table 2). There was no difference in VA between conditions when measured with either technique.

The peak twitch elicited during an MVC (sTw) both by peripheral nerve and transcranial stimulation (Table 2) was unchanged after exercise in the heat, although when normalised to MVC torque sTw increased (p<0.05). However, there was no difference between conditions.

### Corticospinal Excitability

**Figure 5:**
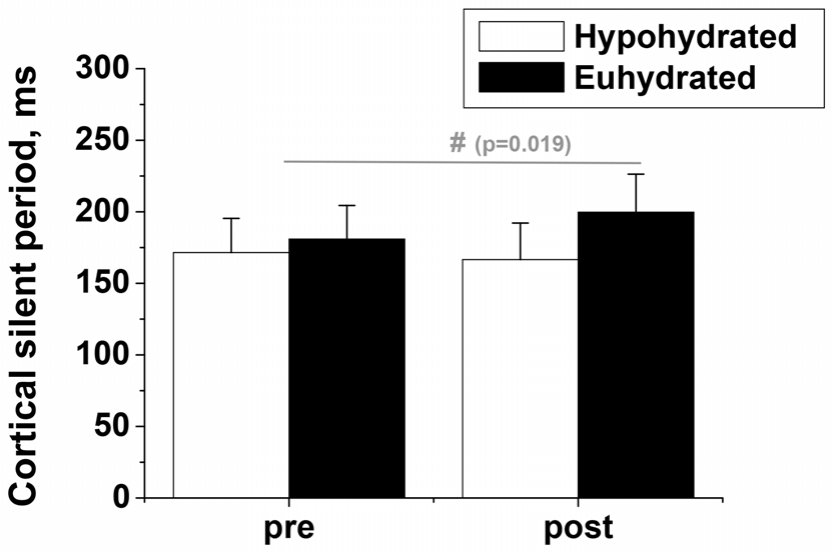
Cortical silent period. Duration (mean ±SEM, n = 9) of the cortical silent period registered in response to transcranial magnetic stimulation (TMS) during maximal voluntary isometric contractions (MVCs) completed before (pre-) and after (post-) 90 min-exercise in the heat in hypohydrated and euhydrated conditions; # - interaction effect.

The normalised resting MEP amplitude (ICC: 0.75) tended to increase after exercise in the heat in both trials (Hy: 85.1±57.4; Eu: 66.1±46.0%; Fig 4C) but this was not statistically significant. During MVC, the normalised MEP amplitude (ICC: 0.73) tended to increase after exercise in the heat in both trials but was not different between conditions (Hy: 25.7±28.5%; Eu: 52.9±33.5%; Fig 4D). The cortical silent period (ICC: 0.91) was lengthened by 10.7±2.4% after exercise in the heat in the euhydrated condition whilst remaining relatively unchanged (−1.8±3.8%) in the hypohydrated condition (p = 0.019, condition × time interaction effect; Fig. 5).

## Discussion

The main findings of the study are that isometric and isokinetic maximum force production were impaired after 90 min exercise in the heat, and these performance deficits were exacerbated in the hypohydrated condition with significantly greater reductions in maximum isometric and isokinetic eccentric force. This additional strength deficit occurred despite significant increases in peripheral excitatory output (Msup) in the hypohydrated condition and appears therefore to be primarily a consequence of reduced peripheral contractility as evidenced by the increased half relaxation time and tendency for slower relaxation rate in the hypohydrated condition. In common with Del Coso et al [13], the hypohydration induced reduction in strength could not be attributed to greater reductions in voluntary activation. Corticospinal excitatory output also tended to increase to a similar extent in both conditions and could not therefore account for the additional performance decrement in the hypohydrated condition. However the cortical silent period, which is suggested to reflect long interval intracortical inhibition, was significantly lengthened in the euhydrated but not in the hypohydrated condition suggesting that hypohydration modulates the excitability of the intracortical circuitry.

The differential effects of hypohydration on peak force production during different contraction modes (isometric, and concentric and eccentric isokinetic), support the conclusion that impaired peripheral contractility underpins the reduction in performance in the hypohydrated condition. The muscle wisdom hypothesis would suggest that the reduction in twitch relaxation rate and increase in half relaxation time evident during the maximal contractions in the hypohydrated condition in the present study will be accompanied by lower firing frequencies of the active motor units in the VM muscle motoneuron pool, a leftward shift in the force-frequency relationship and/or greater motor unit recruitment in response to increased neural drive [26]. Increases in isometric force production above 80–90% MVC are thought to be achieved mainly by increasing motor unit firing rate and synchronisation [27], [28]. Similarly, force development during eccentric muscle contractions is suggested to be more dependent upon rate coding [29], whereas concentric force development appears to be more dependent upon motor unit recruitment [30]. The peak isometric and eccentric torque, which were impaired by hypohydration are reliant upon rate coding; whereas peak concentric force production, which is more reliant upon motor unit recruitment was not significantly reduced by hypohydration. This therefore supports our conclusion that impaired peripheral contractility is the predominant factor in the hypohydration-induced deterioration in performance.

In the hypohydrated condition peripheral excitability increased after exercise in the heat (Msup), in parallel with a tendency for a greater increase in estimated resting twitch (evoked by transcranial magnetic stimulation) and potentiated resting twitch (evoked by peripheral nerve stimulation) in the hypohydrated than euhydrated condition when normalised to MVC torque (Table 2). Although there was no change in the absolute twitch torque produced after exercise in the heat in either condition. However, muscle relaxation was impaired to a greater extent evidenced by extended half relaxation time and slowed relaxation rate (Fig. 3). Perturbations in excitation-contraction coupling therefore seem to be the most likely cause of the greater deterioration in muscle function in the hypohydrated condition. This is presumably related to reduced/slower Ca^2+^ reuptake and Na^+^ K^+^ exchange and hence prolonged membrane re-polarisation. Green et al. [31] recently found that 2 h cycling at 60% VO_2_peak in a thermoneutral environment, resulted in both impaired release and uptake of Ca^2+^ in the sarcoplasmic reticulum. Although no change in Na^+^-K^+^ ATPase activity was reported by Green et al. [31], this has been observed in other studies [32]. No data are currently available on the effects of hypohydration and exercise in the heat on ion exchange, but Hackney et al [33] recently demonstrated that knee extensor muscle volume was significantly reduced by ∼4% when a 3% hypohydration was induced by cycling in the heat with no fluid replacement. This was suggested to be due to sequestration of intracellular water to the extracellular space. In contrast muscle volume was preserved when fluid was consumed during exercise, albeit that in this study the fluid volume consumed was larger (150% of body weight loss) than in the present study (100% body weight loss). One might extrapolate therefore that in the present study an augmented intracellular fluid loss in the hypohydrated trial might lead to greater perturbations in ion balance between intracellular and interstitial compartments and certainly contribute to the impaired excitation-contraction coupling and ultimately to greater force loss.

The decrease in force production caused by prior exercise in the heat in both conditions, and the additional reduction in maximum isometric and eccentric isokinetic force induced by hypohydration could be due to modification at any or all of the levels from somatosensory cortex, motor cortex, corticospinal tract, spinal reflexes, neuromuscular transmission, muscle fibre membrane excitation and action potential conduction to excitation-contraction coupling. The techniques used in the present study do not allow discrimination of the involvement of each of these levels. However, by comparing the transcranially evoked potentials to the compound motor action potential evoked by stimulation of the femoral nerve at rest (Mmax) and during maximal voluntary contraction (Msup), it is possible to isolate alterations in the peripheral excitability from changes in corticospinal excitatory output. After exercise, Msup decreased in the euhydrated condition but increased in the hypohydrated condition yet despite this differential change the isometric torque declined in both conditions and to a greater extent in the hypohydrated condition. This indicates that, despite the increased peripheral excitability in the hypohydrated condition, the voluntary drive was not able to induce equivalent sarcolemmal action potentials after the fatiguing exercise in the heat. The greater reduction in maximum force capacity experienced in hypohydrated condition seems most likely to be due to excitation-contraction coupling failure.

Passive [2], [14], [15] and active [12], [13] hyperthermia with and without hypohydration have consistently been shown to induce central fatigue using electrical stimulation of either the nerve or direct percutaneous stimulation of the muscle or with transcranial magnetic stimulation. Morrison et al. [2] also demonstrated the reversibility of the effect with cooling, showing that voluntary activation and knee extension maximum isometric force production returned to baseline values once the body core was cooled back to normal resting temperature. It is perhaps not surprising therefore that there was no statistically significant effect of exercise in the heat upon voluntary activation (measured using PNMS, Table 2) during maximum voluntary contractions performed in the present study (∼−2%) since core body temperature had returned to resting levels when the post-exercise measures were taken (Fig. 2). Similarly, Del Coso et al. [13] found that the reduction in voluntary activation induced by hyperthermia and a 3.8% hypohydration as a result of exercise in the heat, was not counteracted by water consumption to maintain euhydration. Collectively this suggests that hypohydration *per se* does not exacerbate the reduction in voluntary activation. However, perhaps if we and others had elicited a greater degree of hypohydration, sufficient to compromise health, then decrements in voluntary activation may have emerged. Certainly there was some evidence of intracortical circuitry involvement in the modulation of excitatory output with inhibition significantly increased in the euhydrated condition but not the hypohydrated condition (Fig. 5). This implies the use of different neural strategies in the two conditions in an attempt to preserve motor output. A possible limitation to the extrapolation of this data to the general population is that the TKD athletes participating in this study were well-accustomed to using short term dehydration to make weight for competition. Thus the magnitude of hypohydration effects observed in this study may have been attenuated by the participants' previous repeated exposures, but such a hypothesis needs to be directly tested.

In conclusion, different neural strategies seem to be adopted to regulate neural drive in the two conditions, with increases in inhibitory input of either intracortical or corticospinal origin during the euhydrated trial. Such changes were absent in the hypohydrated condition, yet voluntary activation was similar to the euhydrated condition, perhaps due to smaller increases in excitatory drive rather than increased inhibition. Despite this maximal isometric and eccentric strength were impaired in the hypohydrated condition. The increase in peripheral muscle excitability evident in the hypohydrated condition was not sufficient to preserve performance in the face of reduced muscle contractility or impaired excitation-contraction coupling.
